# Improving Care for Elders Who Prefer Informal Spaces to Age-Separated Institutions and Health Care Settings

**DOI:** 10.1093/geroni/igz019

**Published:** 2019-07-22

**Authors:** Stacy Torres, Xuemei Cao

**Affiliations:** 1 Department of Social and Behavioral Sciences, University of California, San Francisco; 2 Department of Sociology, University at Albany, New York

**Keywords:** Ageism, Aging in place, Community, Isolation, Neighborhoods, Senior centers, Social networks, Social support, Third places

## Abstract

**Background and Objectives:**

Despite advantages of urban areas (such as walkability, public transportation, nearby shopping, and health care services), challenges remain for elders aging in place to access care. The changing demographics of older adults, with higher rates of divorce, singlehood, and childlessness, often living alone and far from family, necessitate new strategies to support health and well-being.

**Research Design and Methods:**

Drawing on 5 years of ethnographic fieldwork and 25 interviews with elders in New York City, this study presents empirical insights into older adults’ use of “third places” close to home, in conjunction with more formal settings.

**Results:**

This article identifies external and internalized ageism and complicated age-based identity as important reasons why older adults preferred “third places” to age-separated spaces such as senior centers and formal settings such as health care settings. We find that neighborhood “third places” offer important physical venues for older adults to process negative or hurried interactions in other formal and age-separated places.

**Discussion and Implications:**

This article makes policy suggestions for increasing access and usage of essential services, including developing attractive and appealing intergenerational spaces in which older community members can obtain services and dispatching caseworkers to public spaces where elders congregate. Furthermore, this article recommends improving exchanges between health care providers and older adults so that they feel recognized, respected, and cared for, which can improve health care outcomes.

**Translational Significance:**

This study’s findings may translate to implementable improvements in the delivery of medical and social services to older adults who live alone and avoid institutional settings in favor of public neighborhood spaces where they can access informal care and social support.

## Background and Objectives

The majority of older adults prefer to age in place. An AARP poll of people aged 50 and older found that 77% want to remain in their community and 76% want to stay in their residence for as long as possible (AARP, 2018). While elders want to remain independent, a growing number live alone. The Pew Research Center finds that 12.1 million, or 26%, of adults aged 65 years and older live alone in the United States (Stepler, 2016). The changing demographics of older adults, with higher rates of divorce, singlehood, and childlessness, often living far from family, necessitate new strategies to support health and well-being. Access to high-quality social and medical services is crucial for elders’ ability to age in their communities. However, they often receive these services in organized settings permeated with ageism, reducing their willingness to participate. Meanwhile, emphasis on organized spaces obscures the importance of public and informal spaces in helping elders age in place.

Baby Boomers have evolving service needs and preferences for maintaining community connections. Despite advantages of urban areas such as walkability, public transportation, nearby shopping and health care services, challenges remain for older adults to access care. Practical and emotional support allows people to age safely and comfortably in their communities. Even when services exist, studies have found that older people do not always take advantage of them. For example, participation in the Supplemental Nutrition Assistance Program (SNAP) is much lower among older adults due to confusion about eligibility, difficulty enrolling, and stigma (Fuller-Thomson & Redmond, 2008).

Drawing on 5 years of ethnographic fieldwork among elders in New York City, this study presents empirical insights into neighborhood-based support for older people. Often research participants did not want to spend time in institutional, age-separated spaces. They used public spaces for health-related support and information when health care and social service settings disappointed or confused them. What hurdles do elders face in elder-serving spaces? How do they use “third places” in conjunction with settings where they receive services? This article identifies ageism as an important reason why older adults preferred informal spaces to age-separated places such as senior centers. We aim to understand participants’ preferences and how they used “third places” to process negative or hurried interactions in other spaces that served older adults, mainly health care settings. While medical settings are not age-separated by design, they serve a disproportionate number of elders who use health care services more frequently.

How can we improve care for elders that eschew the institutional spaces that connect them to social and medical services? Understanding these dynamics can promote new practical measures to improve outcomes for care recipients and for their caregivers. This article makes policy suggestions for increasing usage of essential services, including developing attractive and appealing intergenerational spaces in which older community members can obtain services and dispatching caseworkers to public spaces where elders congregate, whether a park bench or fast-food restaurant. Furthermore, this article recommends improving exchanges between health care providers and older adults so that they feel recognized, respected, and cared for. This study’s findings may translate to implementable improvements in the delivery of medical and social services to older adults who live alone and avoid institutional settings in favor of public neighborhood spaces where they can access informal care and social support.

We review relevant scholarship on elders’ interactions in three types of spaces: age-separated institutions, such as senior centers; organized spaces that serve a high proportion of elders, such as medical settings; and informal “third places,” such as coffee shops and fast-food establishments, where older people socialize across the age spectrum. We then describe our study’s methods, present results from the field, outline translational implications, offer suggestions for reaching elders and improving care, and discuss directions for future research.

### Stigma in Age-Based, Age-Separated Settings: Senior Centers

Senior centers serve as a major policy intervention to help elders remain socially active and avoid isolation. However, the number of senior center attendees has declined nationwide (MaloneBeach & Langeland, 2011). Stigma associated with aging and internalized ageism among elders has contributed to this decline. Many elders dislike socializing only with “old” people and prefer intergenerational activities in nonage-specific community centers (Walker, Bisbee, Porter, & Flanders, 2004). Declining participation also reflects the changing needs of Baby Boomers, a generation synonymous with the saying, “Don’t trust anyone over 30” (Zaslow, 2003) and characterized by post-World War II material surpluses, individualized lifestyle choice and emphasis on youthfulness. Baby Boomers identify more with younger generations, and thus negative stereotypes about senior centers filled with frail and lonely elders discourage their attendance (Hostetler, 2011).

Additionally, senior centers have received criticism for attracting limited segments of the older population (Boggs et al., 2017). Some researchers have found higher participation among healthier, younger, economically privileged elders, and not the least advantaged who arguably need services most. This trend means that center services may not reach many frail, disabled, low-income, and racial/ethnic minority elders (Walker et al., 2004). In recent years, many senior centers have also adopted a “successful aging” business model that serves “customers” with services that espouse a self-reliance ideology, potentially exacerbating the lack of inclusivity and diversity (Hostetler, 2011; Weil, 2014).

### Ageism in Formal Care Settings: Health Care System

Older adults mostly receive health and medical services in organized settings, such as hospitals or neighborhood clinics. Since they tend to use medical services more frequently, they face a greater likelihood of negative interactions in a health care system permeated with explicit and implicit ageism (Adams et al., 2006; Hochschild, 1973). Scholars have found that ageism in the health care system lowers older adults’ willingness to seek professional medical help, reducing their access to health supports to age in place (Hurd Clarke & Korotchenko, 2016). Ample literature documents explicit ageist comments by medical trainees and professionals (Chrisler, Barney, & Palatino, 2016). Aronson (2015) describes one incident in which a reputable surgeon asked a medical student which specialty she wanted to pursue. When she answered “geriatrics,” the surgeon laughed and raised his voice while imitating older patients complaining about constipation. Studies also reveal implicit ageism. For example, health providers tend to baby talk with older patients by speaking loudly and slowly, acting overly polite, and repeating simple sentences (Nelson, 2005). These practices cast older adults as less cognitively and physically capable of communicating than younger adults.

Negative stereotypes also lead health care professionals to provide unnecessary assistance to older patients (Band-Winterstein, 2015). Prior research has found that providers interact with elders in ways that make them feel incompetent, such as discussing their conditions with a family member instead of them (Kane & Kane, 2005). Some mental health therapists have expressed reluctance to accept older women clients due to stereotypes about them as over-emotional (Nelson, 2016). Researchers have found some physicians act more egalitarian and responsive to younger patients (Hatch, 2005). Physicians also tend to attribute older adults’ symptoms to age rather than underlying disease (Kane & Kane, 2005), leading to delayed or under-treatment of pain, fatigue, cognitive impairment, depression, and anxiety and over-treatment with excessive tests, procedures, and surgeries (Adams et al., 2006; Ouchida & Lachs, 2015). Elders face an increased depression risk and delayed diagnosis or treatment in part because health care providers may mistake depressive symptoms as natural aging (Centers for Disease Control and Prevention, 2017). Older adults with higher social and cognitive functioning especially find these forms of implicit ageism condescending, disrespectful, and humiliating (Nelson, 2005).

### Support in Informal and Age-Integrated Settings: “Third Places”

While elders receive most social and medical services in formal and age-separated settings, stigma and ageism there lead some to seek support and care in other informal and age-integrated spaces, especially “third places.” Oldenburg and Brissett (1982) coined the term “third place” to describe the inclusive places outside of home (the first place) and work (the second place), such as post offices, drug stores, coffee houses, and taverns. Third places offer “a home away from home” (Oldenburg, 1999:38) and provide a sense of homelike control and ownership through their physical accessibility, casual and decor, and unassuming design. These venues encourage emerging relationships of proximity (neighbors), service (sales clerks and wait staff), and chance (strangers) (Gardner, 2011). Third places also offer a feeling of warmth through their lively, playful, and welcoming atmosphere (Northridge et al., 2016) and a sense of freedom in conversations which often include gentle joking, teasing, and other expressive behaviors (Oldenburg, 1999). Gatherings in third places are voluntary, informal, and oftentimes anonymous, and thus qualitatively different from more serious and obligatory interactions at home and work (Oldenburg 1999).

In retirement, the second place often fades while the third place becomes more salient, helping elders socialize and structure their days. For elders with limited physical mobility, the proximity of neighborhood third places makes them important physical spaces to get out of the house while maintaining a homey feeling. Rich studies have examined older adults’ interactions in third places. Aside from emerging studies on the “digital third place” (Wexler, Mark, & Oberlander, 2017), a majority examine how elders socialize in “commercial third places,” such as shopping malls, fast-food restaurants, and cafés. For example, many elders do not view shopping centers as consumer hubs but as places to “do nothing” or enjoy “people watching” (White, Toohey, & Asquith, 2015). Due to their safe, lively and comfortable atmosphere, inclusive physical amenities (such as benches), and low or no cost, some elders go to shopping centers exclusively for social contact and interaction (Graham, Graham, & MacLean, 1991). Fast-food restaurants serve as another popular third place for elders. For example, Cheang (2002) observes a group of elders who frequent a Honolulu fast-food restaurant. He finds that this spontaneously-occurring group not only provides structure, meaning and social interaction, but more importantly, facilitates fun and leisure for long-term well-being.

Studies also identify public parks as a “communitarian third place” that provide elders with open space to cultivate social ties. Using still photography in Israel, Noon and Ayalon (2018) highlight the presence of older adults in parks. They find that although most arrive alone, almost half form a group there, suggesting the importance of these spaces in promoting social interactions. Public spaces bustling children, families, workers, and other passersby often help elders build a sense of community and connection (Finlay, Franke, McKay, & Sims-Gould, 2015). Considering that many enjoy multigenerational places more than age-separated spaces, the current study adds to the emerging literature on late life and third places and aims to understand why and how older adults use third places, why some prefer them to more formal and institutionalized places, and how service providers can reach their elder patrons.

## Research Design and Methods

The study draws on 5 years of ethnographic fieldwork and 25 interviews with participants. The initial site, a mom-and-pop bakery, sat at near a co-op for moderate-income residents, public housing projects, and a large rental complex. These buildings housed many longtime tenants, a number of whom frequented the bakery. Their rental protections meant that many lower-to-middle income residents had stayed in a neighborhood now home to luxury apartments and businesses. Many co-op and project residents had “aged in place” in buildings classified as Naturally Occurring Retirement Community (NORCs), which Federal law defines as “a community with a concentrated population of older individuals” (Niesz, 2007). New York City has 27 NORCs in four boroughs (Interboro Partners, 2010).

According to 2015 American Community Survey 5-year estimates, 14% of the neighborhood’s population are 65 years and older, with 75% of those women and 25% men. About 52% of these households have older adults living alone; men make up 40% of these solo dwellers and women 60%. Forty-nine percent of renters and 26% of homeowners over age 65 have lived there for more than 36 years; only 9% of renters and 7% of homeowners over 65 have less than 5 years of residence (United States Census Bureau, 2015).

The data come from a study designed and conducted by the article’s first author. Her experiences as a bakery customer helped her meet other regulars. This group of roughly 47 women and men were aged 60+, the majority of them women, Puerto Rican, Jewish, and ethnic white (Italian, Greek, Irish descent), and low-to-middle income. During a 6-month period, she observed the bakery during morning, afternoon and evenings an average of four to five times a week for a minimum of two hours. She recorded her observations until the bakery closed due to the owner’s inability to renew the lease for a higher monthly rent. She identified five sites where she hypothesized former customers would go, based on their plans to cope with the closing, housing proximity to the original site, and knowledge of the neighborhood. She ended up primarily in two sites for four and a half years—McDonald’s and Pete’s Delicatessen—where different groups of former bakery patrons had convened.

The first author’s entry into the field began with a project for an ethnographic research course during her doctoral studies. She received approval from the Institutional Review Board to conduct participant observations and interviews. To potential participants she explained that she wanted to learn more about how older people age in place by spending time with and interviewing residents over age 60. Participants permitted her to record observations of their interactions and a subset participated in a digitally recorded interview. She explained her efforts to protect confidentiality, making sure participants understood that participation was voluntary and that they could choose to participate in parts of the study and not others (e.g., ethnography and/or interviews) and withdraw any time. We have changed the names of participants and most locations to protect confidentiality.

The first author took handwritten field notes at the sites and afterwards. She uses quotes when she wrote down in her notebook what people said as they spoke or shortly afterwards and when she transcribed dialogue from tape recordings made in the field or during an interview. Speech in quotes should be taken only as a close approximation. After writing detailed notes of the day’s observations and conversations, she added brief analytic notes and memos which helped later to discover patterns, prompt additional questions, and make connections between data and the literature. Analysis consisted of manual reviewing fieldnotes and interview transcripts for relevant themes and emerging patterns, coding for salient concepts and categories generated from reading the literature and the data.

The number of people observed is higher than the core group of regulars with whom the first author spent the most time and totaled approximately 136. She also visited participants in their homes, hospitals, nursing homes, and accompanied them to other neighborhood places. To supplement observations, she conducted 25 interviews with people recruited from the bakery. These interviews asked about basic biographical information, residential history, experiences of the bakery and afterwards, social relationships, and daily routines.

## Results

### Senior Centers: Resisting Age-Based, Age-Separated Institutions

While a few participants regularly attended one of the neighborhood’s four senior centers, including one woman who traveled to the state capital to protest budget cuts, most avoided them and many expressed their dissatisfaction. “I hate old people. And I don’t want to sit there all day and play mah jong, or whatever they do,” Eugene offered as an explanation for his dislike of senior centers. He chuckled afterwards. Though he claimed to hate older people, at 90, he spent most of his free time with similar aged peers. But his desire to distance himself from other elders, or at least the less desirable company he expected in an age-separated, institutional space, offered a glimpse into the internalized ageism common to many older people (Cruikshank, 2009) and prevalent among research participants. Sylvia also expressed negativity about senior centers.” That’s not for me,” she said. When prodded, she mentioned attending a hospital-based support group after her husband’s death nearly two decades before, “I went once and never again; it was the most depressing thing ever.” She viewed senior centers as similarly “depressing,” though she had never visited one because she felt people who “needed help” went there. Eddie took advantage of a monthly MetroCard bus parked outside one center, which spared him having to navigate the stairs in the subway to add money to his discounted transit pass. But he avoided the center’s other social activities.

Lucy also expressed reservations about senior centers, not due to internalized ageism but because of her resistance to identifying with other elders solely based on age. A never-married, single woman, she spent a lifetime creating relationships and a daily routine that kept her busy and out of the house. A retired secretary approximately in her late 70s, Lucy often complained about getting older. “Getting old sucks!” she said. She also spoke of feeling worn out and difficulty filling her excess free time. Since retirement Lucy had shifted her time, attention, and energy from the demands of paid work to caring for herself and her body. Contrary to her lamentations about boredom, she packed her day with activities such as visits to the bakery and McDonald’s, grocery shopping, dinner with friends, and the occasional theater performance or movie. Less palatable errands such as her roster of medical appointments also helped structure her day.

Lucy had expressed her financial stresses often when she gathered at the bakery, Pete’s, and McDonald’s. Much of her financial misery related to rising health costs and confusion about her medical plan’s benefits. She often brought medical bills and notices from Medicare to show other regulars. In 2010, she worried about the impending rollout of the ObamaCare health plans and changes to her Medicare plan. Lucy spoke often about this perceived threat with another older woman named Vivian, who also expressed concern and anger at President Obama.

One evening in Vivian’s absence, Lucy recounted how Vivian had urged her to organize around Medicare with the women who attended the senior center in her building. With anger she explained: “You know what she wanted me to do…she said, ‘You should go down to the senior center in your building and get some of those women to protest with you.’ How do you like that? She wants me to protest. She wants me to do the work. Well, I told her she should go over there and protest. Those women are not like me. They were married and had children. They didn’t work. They go down there and knit.”

Though she disliked Vivian’s pressure, Lucy’s objection dwelled in her resistance to identify with other older women on basis of age alone. Despite sharing membership in a 65+ age group, residence in a building with many older adults, gender, and race (white), Lucy resisted recognition and instead focused on their differences. Lucy’s lack of identification with these other senior women shows that age-based identification is complicated even when the opposition does not stem from a desire to delineate hierarchy but from a lack of recognition due to divergent life histories. Such diversity in life experiences means that age-based and age-separated spaces such as senior centers face difficulties satisfying the range of elders’ needs. Service and health care providers must carefully consider their heterogeneity when designing spaces and programs for seniors. A few years after this incident, Lucy mentioned how much she enjoyed attending a senior center focused on serving sexual minority elders, though she did not openly identify as LGBTQ+.

### Health Care Settings: Dissatisfaction and Resignation

Visits to health care sites loomed large in most participants’ lives, especially those with multiple chronic illnesses. Some received primary care at one of the two clinics located a few blocks from their homes, including one specializing in geriatric medicine. But many also traveled outside of the neighborhood for care, especially when needing to consult with a specialist. When someone returned from a medical appointment, regulars often engaged in the after-appointment analysis. These interactions provided a chance to vent, compare, and share medical problems. After Eugene returned from the Veterans Affairs (VA) hospital’s geriatric clinic, along with medical updates, he always came armed with stories of how the staff condescended to him. His accounts gave people opportunity to give their opinions about his treatment and share their experiences. He voiced his distaste for the protocol that staff followed to assess his physical status and mental acuity. They asked if he could tell them the day of the week and explain how he traveled to the clinic on his own. Once they took their assessment further, removed Eugene’s shoes, and asked him to demonstrate that he could put them back on without assistance. Afterwards Eugene wrote a complaint letter to the VA hospital director and received a written apology.

“When I go to the doctor, it’s always the same thing, ‘What day is it, here are three objects, an apple, a dog, an airplane,” Eugene imitated in a sing-song voice. “Then they walk away and quiz you later. Do you know how many times I’ve drawn a clock with its hands at ten of twelve?”

“Oh yes, I hate that one…when they leave you sitting there and walk away,” Sylvia said. “Uh huh, and what if you forget one of the things? What then?” she shuddered. “And the other one, where they write the letters, that’s a tough one.” She described an assessment in which a doctor instructed her to close her eyes while he traced an alphabet letter on her open palm, then asked her to identify the mystery letter.

“Well, here’s the deal,” Eugene continued. “What I don’t understand is, if they want to make sure I’m not senile, why don’t they discuss current events with me, ask me what’s going on in the world or if I’ve read the newspaper? I’m the author of over fifty books.”

Sylvia confessed that these “tests” made her nervous and that she too hated them, “But what choice do I have? They need to check for certain things.”

Eugene rolled his eyes. “Well, I don’t have to take it,” he said.

“I don’t like to make waves,” Sylvia cautioned. She was a vocal proponent of following doctors’ orders, even if skeptical. She also encountered ageism during medical appointments and claimed that elders received dismissive treatment, “When they see that number on the form, when you get above a certain number.” When she spoke with Eugene about his health, her voice betrayed concern. She sounded fearful of what could happen to him if he argued with the VA staff, as when he refused to take his medication after his heart attack. Sylvia counseled him to accept the treatment he detested, acknowledging its less than ideal qualities but arguing that they must follow doctors’ recommendations if they wanted to live. Eugene and Sylvia rarely agreed. He admitted to postponing and cancelling appointments to avoid interacting with clinic staff. He described his latest negative experience.

“They showed me pictures of a giraffe, a rhinoceros, and a lion, and asked me to name them. Can you believe that?” he said.

Eddie shrugged after listening to Eugene’s frustrations. “Look, it’s the geriatric clinic. This is what they do to everyone,” he said with resigned acceptance from his own experiences receiving care at the VA hospital. “It is what it is, when you get up there in years.”

Eddie’s acquiescence reflected his typical response, to suggest that Eugene’s doctors were looking out for his best interests.

Eddie and Eugene offered a potent comparison in their approaches to accepting medical intervention, keeping appointments, and adhering to medication regimens. Despite his blustery demeanor, Eddie vigilantly took every pill and test that his doctors prescribed. Eugene often claimed to know more than his doctors. He used past negative experiences, such as a bad side effect or a doctor’s error, to justify his selective approach to dealing with the medical establishment. Though Eugene had a more genteel air, he resisted accepting without question anything his doctor recommended. He felt that he had sufficient expertise, due to his education and writing on medical topics, to evaluate and dismiss parts of the treatment plan.

Eddie, Sylvia, and Lucy acted most traditionally responsible about their medical care. They attended doctors’ appointments and followed orders without much modification, despite hurdles to complying with a prescribed treatment, such as high out-of-pocket costs. Their compliance did not mean they never complained about aspects of their regimens. But after discussing their concerns or questions with family, friends, and assorted neighborhood people, they either consulted with their doctors and agreed to abide by their orders or otherwise reconciled themselves to the treatment.

Others were less accepting. They took only some prescribed medications and attended those appointments and medical tests deemed necessary. In the morass of health care services, a few identified bright spots. Sylvia pointed to her physical therapist and Lucy to her acupuncturist as attentive exemplars. They described these providers’ patient listening and spending time with them to understand connections between symptoms. They treated the body as a system rather than a disconnected series of parts, unlike some specialists. Eugene remained ambivalent. He did not avoid doctors altogether but modified his regimen and lapsed in the continuity of his care, such as when he got frustrated with the condescending treatment at the VA hospital and postponed and cancelled appointments until they refused to refill his medication. Yet, despite his resistance, consulting with others in public places helped him comply with more of his medical regimen due to subtle peer pressure than he might otherwise if he had nowhere to discuss his dissatisfying experiences.

### Support in “Third Places”

For older adults living alone, third places served as important physical sites to receive informal care and advice as they shared struggles and helped others decode information received at the doctor’s office. Lucy had a history of arthritis and temporomandibular joint disorder, which causes chronic pain in the joints and muscles responsible for jaw movement (Mayo Clinic, 2018). Despite claims of fatigue and chronic health issues, her body allowed her to maintain her routines and social interaction in the neighborhood.

When Lucy finally arrived at Pete’s in the early evenings she inventoried a typical day’s comings and goings, including gossip from McDonald’s, updates from her doctor’s appointments, and other daily frustrations. She described the minutiae of her life with exquisite detail: haggling with cable customer service representatives about a faulty remote; disputing billing errors with her health insurance company; resolving a problem obtaining a prescription at the pharmacy. “I used to be a fighter,” she said. Her penchant for hyperbole not only reflected her distress but also evinced a larger observation about late life, insofar as many research participants discussed their daily experiences as depleting, requiring energy they no longer had, and therefore a test of physical and emotional endurance.

Often Lucy’s tales involved a show-and-tell component. She reached in her purse and surprised us with the latest medical or assistive device she had purchased. Sometimes she displayed the item for us to admire or to solicit our opinions. Other times she needed help figuring out how to insert batteries or demystify the directions. One evening she brought a leftover acupuncture needle from an earlier appointment. Another occasion she showed us a pendant on a chain designed to sound an alarm and alert her friend to an emergency like a fall in her home. Once she had difficulty fitting a dental guard for jaw pain over her teeth but with effort positioned it correctly, while Sylvia and Eugene watched. Sometimes Lucy purchased these items upon a doctor’s recommendation, but mostly she had determined that they might mitigate a problem.

Lucy experienced many health problems common to older adults, such as worsening arthritis. But they had not yet impeded her ability to circulate throughout the neighborhood and chat with patrons at local businesses. Ultimately a series of falls proved the biggest challenge to her autonomy. She fell one afternoon at the supermarket while standing still, not due to an external hazard such as a cracked sidewalk or snagged carpet.

“I just went down,” she admitted. A month later she fell again at home.

After her first big fall she began using a cane, which guarded against the potential catastrophic consequences of future falls and strengthened her sense of security. Geriatricians have long identified the fear of falling in older adults as a public health problem linked to an elevated fall risk (Scheffer, Schuurmans, Van Dijk, Van Der Hooft, & De Rooij, 2008).

“I felt like I was gonna die,” Lucy repeated often.

Despite her falls Lucy continued to go to Pete’s and McDonald’s. Following a major snowfall we missed her at Pete’s for a few days. But as winter eased, Lucy still stayed closer to home. Instead of heading to Pete’s around 6 p.m., as she had in preceding years, she remained at McDonalds’s. Pete’s stood three and a half blocks away from her home whereas McDonald’s came in at a single cross-town block. Eugene’s camaraderie was not enough to lure her to Pete’s, though she showed up occasionally, with several days and sometimes a week between visits. This drop-off in her appearances surprised people, given the steadfastness of her presence over the years and her fondness for Eugene’s company. She often stayed at Pete’s with Eugene for hours after everyone else had left.

Lucy’s final fall resulted in a broken ankle. After several weeks she returned to Pete’s but never regained her prior levels of attendance. She mentioned getting depressed when stuck in the house, with no need to put on makeup or dress well. Usually she colored her hair at the salon every few weeks, varying the shade from dark honey to blinding platinum. Her frequent references to that period showed how profoundly the bad weather and injury had affected her.

“I didn’t see anyone. I had no reason to do myself up,” Lucy said.

“And you’re usually dressed to the nines,” Eugene said.

“Yeah, I know,” she agreed glumly.

Though Lucy endured greater isolation, she soldiered through that winter. We saw her regularly despite gaps between her outings, and she continued to rely on neighborhood venues to vent her worries about her new physical problems and to commiserate about the stresses of the winter weather.

For those that withdrew from health care settings altogether, third places arguably provided even more support. Dottie’s growing health problems drew attention as she had trouble walking more than a block. Her neighborhood contacts voiced concern and criticism of her weight. But unlike another regular named Joan, who spoke of avoiding doctors for years, Dottie hid her avoidance from friends, family, and neighborhood acquaintances. Before her primary care physician retired, Dottie referenced seeing him regularly at his office two blocks from her home. The only other doctor she mentioned visiting was her longtime podiatrist, Dr. Gurvits, whose office stood across the street from the housing projects where she lived. She remained under his care until he also retired. They had built such a close relationship that he gave her a copy of his apartment keys in case she ever needed to stay with his family in an emergency.

Dottie never informed the regulars at her neighborhood hangouts about her lapse in medical care. After her heart attack and subsequent hospitalization her daughter Linda said that she had not seen a doctor besides her podiatrist for years. Dottie remained in the hospital for 4 weeks, followed by a 2-week stay in a nursing home, before she died at 83.

“The only person she was seeing was Gurvits,” Linda said, when asked if her mother told her she had seen other doctors.

“Lies, all lies,” Linda said. “After Gurvits was gone, she didn’t see no one at all. No medication, nothing. She weighed down that cart with a bunch of phone books,” she added, clarifying how Dottie had managed to fashion her cart into a makeshift rolling walker to navigate a two-block radius around her building, as her mobility worsened. She said she had offered to accompany her mother to doctor appointments but Dottie had declined. Despite these gaps in care, Linda pointed to one reliable presence in Dottie’s life. “I always knew where I could find Mom,” she said, referring to the bakery.

## Discussion and Implications

As growing numbers of older adults live longer, they will need to seek a range of support for health-related challenges. Our findings suggest more will rely on company in informal spaces as a supplemental, and in some cases primary, form of support as they age in their communities. In line with prior scholarship that has found ageism in health care settings (Kane & Kane, 2005), greater mistrust of the medical system among racial and ethnic minority elders (Pan et al., 2014), and dislike of age-separated institutions like senior centers among a high proportion of older adults (Walker et al., 2004), these findings suggest increased need to understand elders’ options for alternative spaces and further empirical research on how elders reach beyond formal settings for assistance to remain healthy and independent.

These vignettes show how and why study participants avoided or preferred other types of spaces outside those that traditionally serve older adults, like senior centers, and the shortcomings of medical infrastructure. Even those who participated in formal settings had mixed experiences that may have prevented them from taking full advantage of available services. Often participants expressed not feeling listened to by medical staff and resisted identifying with the stereotypes they encountered of older adults as dependent and deserving of pity. They internalized cultural messages about the devaluation of older adults, and these ageist beliefs surfaced in their reasons for avoiding age-separated institutions geared towards elders. Yet, under conditions of their making, despite their dislike of age-segregated settings and hierarchical distinctions made between different older people, they enjoyed the company of similar aged peers.

Elders vulnerable to isolation due to living alone, health and mobility problems, cultivated a rich network of neighborhood connections in public spaces. They consulted each other about health care decisions and frustrations and received practical assistance evaluating their options. Third places provided somewhere to vent and reliable listeners. Underlying narratives of positive and negative experiences with the medical establishment stood the desire to build relationships with providers and wanting to be known, seen, and cared for. This craving for connection stood out as especially important for people living in a landscape of loss later in life.

This study shares the limitations of many small qualitative studies that draw on non-random samples and cannot generalize to the population. We also do not have observations of elders’ stressful interactions at medical offices. Future qualitative research that includes these data would provide additional depth. Despite these limitations, this work expands prior scholarship on elders’ socializing in public spaces and adds to a small but growing number of qualitative studies on lived experiences of aging in place (for some recent examples, see Abramson, 2015; Loe, 2011; Portacolone, 2013; Rúa, 2017; Weil, 2014). This study provides insight into elders’ desire for community spaces and how they creatively patched together practical and emotional support when their needs went unfulfilled in other venues they found stigmatizing. We can draw translational implications from these observations for practice, policy, and future research.

Given the ageism and stigma attached to organized spaces for seniors, many scholars and practitioners have proposed to modify senior center models to align with older adults’ spontaneous and voluntary uses of public spaces. For example, based on a survey of Baby Boomers in the Midwest, Walker and colleagues (2004) suggest that service providers re-envision senior centers as multipurpose age-integrated centers instead of spaces targeted to elders. Markwood (2013) recommends creating “ageless” activities and services for all ages in addition to programs for older adults. This proposal also moves services outside traditional senior centers and suggests that health education classes could occur in local hospitals, computer and technology training classes in community colleges, fitness classes at recreational centers, lifelong learning classes at libraries, financial planning courses at shopping malls, and virtual online senior spaces available any time. By branding these elder-serving spaces “intergenerational community centers,” researchers expect to attract and benefit more older adults (Markwood, 2013).

Among the new generation of programming, Mather’s More Than a Café model (MMC) has gained much positive attention (Gustke, 2016) and adoption by more than 40 organizations across the United States (Café Plus in Action, n.d). The nonprofit café provides a bottomless coffee cup for 95 cents and opportunities for all ages to socialize. They offer dozens of classes to adults over 50, including painting, cooking, flower arranging, strength training, and iPhone basics (More at Mather’s, n.d). They also developed a “telephone topics” program in which older adults call toll-free to participate in an array of activities, such as guided chair yoga or meditation sessions, sports and movie discussions, vocal performances, and storytelling sessions (Telephone Topics, n.d.. Evaluation studies of the MMC programs suggest that participating seniors experience mental restoration from fatigue, loneliness, and difficult life events such as death, divorce, illness, and retirement (Rosenbaum, Sweeney, & Windhorst, 2009; Rosenbaum, Ward, Walker, & Ostrom, 2007). The retirement community Newbridge on the Charles has also received acclaim for their multigenerational programs (Gawande, 2014). By bringing together senior residents and elementary to college-level students, programs disrupt barriers between old and young, bringing companionship to elders and helping students understand elders’ experiences and needs (Intergenerational Programming, Hebrew SeniorLife, n.d).

Other than designing age-integrated and intergenerational programs, we can also create more age-friendly public spaces for elders who seek care options in informal spaces, such as parks, diners, and shopping malls. Local governments can improve the quality of urban spaces by incorporating “green space” and “blue space,” such as gardens and courtyard fountains (Finlay et al., 2015). We also recommend installing comfortable street furniture (chairs, chess tables, etc.), mending potholes, maintaining free-of-obstruction pavement, and building clean public toilets to make streets more accessible (Noon & Ayalon, 2018; World Health Organization, 2007). Businesses can also help cultivate spontaneous and supportive relationships among customers by installing comfortable and accessible seating and allowing customers to linger (Rosenbaum et al., 2007). Community-based organizations can also host affordable or free events connecting health services to older adults who frequent neighborhood businesses or public spaces, thus helping medical professionals and social service workers to reach underserved older adults who spend more time there. Indeed, health professionals have already recognized the importance of third places in elders’ lives and offered health services in these sites. For example, Northridge et al. (2016) detail how the ElderSmile program at Columbia University College of Dental Medicine has reached older adults in third places for preventative oral health care services, and how older adults who received services later helped recruit additional participants for other program activities, including dental, diabetes and hypertension screenings.

We recognize the value of elder-serving institutions, despite their shortcomings. We suggest improving these age-separated spaces so that elders find them more attractive and inviting, but without rebranding them in ways that alienate or abandon their essential purposes, such as providing free or low cost nutritious meals (Weil, 2014). Additionally, reducing bias and improving interactions between medical providers and elder patients is a paramount concern. Following Nelson’s (2005) suggestions, we recommend policy to mitigate age bias in the medical system, including increasing medical professionals’ awareness of their ageist beliefs, instituting antiageism training programs early in medical education, and expanding geriatrics programs in hospitals and mental health offices.

Future generations of older adults will have greater diversity. As the current study uses ethnographic data collected from fieldwork with a group of mostly ethnic white and Puerto Rican, poor to middle-income elders who identify as heterosexual, future studies should consider variations in experiences of aging in place among racial and ethnic minority, immigrant, and LGBTQ+ elders. As Pan, Stutzbach, Reichwein, Lee, & Dahodwala (2014) show, compared to Whites, African American elders have greater mistrust of the health care system, while Chinese-American elders report more language difficulties accessing health care. Older racial minorities also often use alternative health care, such as herbalists or music for self-care (Hansen, Hodgson, & Gitlin, 2016). How do they perceive the health care system? Are they underserved in these settings? Where do they socialize and find connection and social support? What beneficial health practices do elders in these communities use? Lastly, how shall we design public spaces to meet the needs of diverse elders so that future generations may receive innovative care in the community?

## Funding

This project was funded in part by fellowships from New York University, the American Sociological Association Minority Fellowship Program (cosponsored by Sociologists for Women in Society), and the Ford Foundation.

## Conflict of Interest

None reported.
